# Learning From Highly Effective Teams: What Can We Apply to the Gastrointestinal Endoscopy Unit Team?

**DOI:** 10.14309/crj.0000000000000745

**Published:** 2022-02-24

**Authors:** Nicholas M. McDonald

**Affiliations:** 1 Division of Gastroenterology and Hepatology, University of Minnesota Medical Center, Minneapolis, MN

## INTRODUCTION

A team is defined as 2 or more people interacting dynamically and adaptively toward a common goal, with each member filling a specific role within it for the group to function as a cohesive unit.^1,2^ Taskwork represents a task an individual team member is doing, whereas teamwork represents how teammates perform the task in relation to each other.^3,4^ By joining a team, individuals stand to benefit from the collective knowledge, experience, or skills of the group while working toward a common goal.^5,6^ Highly effective teams commonly are focused on a specific goal and, through collaboration, can achieve results at a rate that is higher than the sum of the contributions of each member as individuals.^6^ As part of a sports team, the goal might be to win a championship. In the business world, the goal might be to generate a target revenue. In a gastrointestinal endoscopy unit, the goal is to deliver safe and effective patient care. When applied to this setting, a single professional rarely delivers healthcare alone, and optimizing teamwork is critical.^7–9^ Within the endoscopy unit, the stakes are especially high because poor teamwork in endoscopy can lead to medical errors or near misses.^7,10^ Conversely, a strong collaborative team can take on medical complexity, acuity, and high procedural volume while functioning at a high level. We can learn and apply habits from sports and business to help achieve strong collaborative teams in the endoscopy unit.

## HIGHLY EFFECTIVE TEAMS IN SPORTS

Many remember momentous sporting events resulting in upsets where an underdog, heavily favored to lose, claimed victory. Whether it is the memorable “Miracle on Ice,” where the United States defeated the Soviet Union in the 1980 Olympic hockey game or smaller scale matches such as community sporting games, it is not uncommon to see athletic teams perform at a higher level than one would expect based on the sum of their parts.^11^ Although a sports team requires members to have appropriate knowledge, skills, and abilities, there are many more components that go into making a highly functioning and effective team.^12^ One study by Webster et al^12^ conducted interviews of 21 players, coaches, and sports psychologists to identify characteristics of highly effective teams in elite-level cricket. Open and honest communication along with clearly defined roles was especially critical to a highly functioning team.^12^ Another key finding was the importance of effective leadership, not only from the coach but also from the team captain leading peers.^12^ In the setting of a highly effective cricket team, the coach is responsible for being a positive role model and having a clear vision and direction for the team, whereas the captain's role is to lead by example and to motivate and inspire others.^12^ Although not every quality of a highly effective sports team translates to an endoscopy unit team, the need for clear communication, defined roles, and effective leadership translate well to ensure the best outcomes possible.

## HIGHLY EFFECTIVE TEAMS IN BUSINESS

One of the major goals of an effective business is to generate the maximum amount of revenue while minimizing cost.^2^ A highly effective team in business is one where individuals can minimize costs and generate resources at a level beyond what is expected.^2^ In addition, an effective team requires appropriate team task analysis which entails choosing the proper roles for individual team members that best suit their unique skillsets and abilities.^2^ If a team assigns a member to perform an accounting task, but the individual lacks the necessary skills, then it will be unlikely for that team to be highly effective. Thus, it is important to choose an appropriate team composition.^2^ If composition assessment is poor and the resulting team has too few members or not enough members with the appropriate skills, a team is unlikely to succeed in this sector. If a business team member does not have the necessary skills, they might receive targeted feedback, additional training, or the business might hire someone else with the required skill. Alternatively, if there are too many team members or overqualified members, they should be optimally allocated to minimize overall cost and optimize productivity. Although the goal in an endoscopy unit team is patient care, rather than purely financial output, many of these ideas from business still translate to an endoscopy unit team.

## APPLICATION TO GASTROINTESTINAL ENDOSCOPY

Although qualities that help a business team achieve success may be different from those that help a baseball team succeed, traits from each can be applied to teams in endoscopy. Several traits were common across teams in multiple disciplines, including communication, effective leadership, adaptability, and well-defined roles. Communication is one skill which is found in many highly effective teams across various disciplines.^4^ For the endoscopy unit team to be highly effective, it is vital for communication between team members to be effective and avoid communication that is either ineffective or destructive. One application of effective communication for an endoscopy unit team is closed-loop communication. Closed-loop communication is a 3-step process to ensure effective and clear communication.^13^ The first step is transmitting a message to the intended receiver and using their name when possible, such as a physician asking a technician to obtain a snare. In the second step, the receiver acknowledges and confirms hearing the correct message and asks for clarification if needed, such as “I am obtaining a snare, what type of snare would you like?” Finally, the original transmitter verifies the message has been correctly interpreted and closes the loop, “Thank you, that is the correct snare.”^13^ Although it may seem simple, closed-loop communication can ensure clear and effective communication and can help endoscopy unit teams be highly effective.

For a highly effective business team, team task analysis or choosing the right person with the right skills for the job is critical. This translates to the endoscopy unit team because it is essential that we identify what role each team member should play and the skills they will need to be successful in that role. If a gap is identified, such as a technician not having experience with a tool, then teaching should be offered to help them acquire the skills necessary to be successful. In a healthcare setting, the physician may not always be choosing who their team members are or they may have different levels of experience. Targeted feedback can also give team members the tools to be highly effective. If a team member is lacking specific knowledge, skills, or ability, constructive feedback can help them improve.

Businesses rely on appropriate team composition determination—figuring out how many people are needed for a job. In the endoscopy unit, it is pivotal to determine the number of people and roles needed to be successful. If an endoscopy unit does not identify the appropriate team composition required for a complex endoscopic procedure, then that team may not have enough people in the room when a situation requires it, and the team is unlikely to be highly effective.

In sports, transparent and effective leadership is essential for a highly effective team. It is also imperative to have clear and effective leadership in the endoscopy unit. The role of the endoscopist may be seen similarly to the role of team captain in cricket—role modeling best practices and motivating team members. Many traits of an exceptional endoscopic leader have been previously reported, including a recent article about scopesmanship.^14^ One area where endoscopic units differ from highly effective teams in sports is the lack of a coach. In this regard, endoscopy unit teams may stand to learn from athletics. Although the captain (endoscopist) provides leadership within the case, it is the coach's role to provide high-level oversight and guidance. Atul Gawande wrote, “No matter how well prepared people are in their formative years, few can achieve and maintain their best performance on their own.”^15^ Gawande wrote about the benefits of coaching for elite performers, including surgeons to achieve their personal best. It may be beneficial for endoscopists to consider having a senior or effective colleague play the role of coach by watching the endoscopist perform procedures and giving advice or guidance on improving team effectiveness. In addition, a systematic review and meta-analysis evaluated controlled interventions to improve team performance and found that team performance can be improved through targeted intervention, suggesting that perhaps, teamwork in the endoscopy unit could also be improved through targeted interventions of perceived weaknesses of the unit team.^4^ By understanding traits that are critical for highly effective teams in business or sports and thoughtfully implementing them into our endoscopy unit teams, we may be better able to produce the superior results and outcomes that our patients deserve (Table 1).

**Table 1.** Highly effective qualities applied to endoscopy

Highly effective team quality	Application to endoscopy
Effective communication	Ensure closed loop communication with endoscopy unit staff and solicit feedback on how to communicate more effectively.
Leadership	Endoscopist role modeling best practices and motivation of team members. Consider designating an experienced “coach” to observe interactions and recommend tips to improve teamwork.
Task analysis	Identify skills of staff members and determine what knowledge, skills, and abilities will be required for an endoscopy unit. Ensure team members have the appropriate skills or receive training.
Team composition	Identify the number of staff and roles needed to perform required tasks. Ensure tasks are staffed with the appropriate amount people to be successful.
